# bioORMOCER^®^—Compostable Functional Barrier Coatings for Food Packaging

**DOI:** 10.3390/polym13081257

**Published:** 2021-04-13

**Authors:** Katharina Emmert, Sabine Amberg-Schwab, Francesca Braca, Agostino Bazzichi, Antonio Cecchi, Ferdinand Somorowsky

**Affiliations:** 1 Fraunhofer-Institut für Silicatforschung ISC, Neunerplatz 2, 97082 Würzburg, Germany; katharina.emmert@isc.fraunhofer.de (K.E.); sabine.amberg-schwab@isc.fraunhofer.de (S.A.-S.); 2 Laboratori ARCHA Srl, via di Tegulaia 10/A, 56121 Ospedaletto (PI), Italy; francesca.braca@archa.it (F.B.); Agostino.bazzichi@archa.it (A.B.); Antonio.cecchi@archa.it (A.C.)

**Keywords:** sustainable food packaging, functional barrier coating, bioORMOCER^®^, aminated hemicellulose, oxygen transmission rate, biodegradable packaging, compostability, antimicrobial properties

## Abstract

Biodegradable packaging materials are already in use. However, there are severe restrictions preventing the broad application in food packaging, especially due to insufficient barrier properties. Our idea was to improve these properties with a biodegradable coating. The Fraunhofer-Institut für Silicatforschung ISC has been developing high-barrier coatings for various packaging applications based on a class of materials with glass-like structural units, named ORMOCER^®^. However, these state-of-the-art ORMOCER^®^ coatings are not biodegradable. The aim of our work was to modify ORMOCER^®^ to become biodegradable and, at the same time, preserve the barrier and functional properties. This was achieved by the incorporation of functionalized tamarind hemicellulose Glyate^®^ into the ORMOCER^®^ matrix. For this purpose a two-step amination reaction of Glyate^®^ was chosen. The aminated product was analyzed by FTIR, solid-state NMR and elemental analysis. New aminated Glyate^®^ containing bioORMOCER^®^ lacquers could be synthesized. Lacquer quality assessment was performed by Raman spectroscopy. The properties of the resulting coatings were evaluated by laser scanning microscopy (LSM), oxygen transmission rates (OTR) measurements, E-Module determination and adhesion tests. Standardized tests for compostability, overall migration and antimicrobial properties were performed for the bioORMOCER^®^ coatings. The evaluation showed that the new bioORMOCER^®^ coatings are suitable for sustainable food packaging.

## 1. Introduction

Nowadays, fresh food as well as convenience food reaches customers usually packaged. Hygienic conditions, long shelf life and easy availability of these packaged products in almost every moment of daily life account for a large share of our living standard. Flexible packaging is the most economical method to package, preserve and distribute food, beverages, consumables, cosmetics, pharmaceuticals and other products that need extended shelf life. According to a market report by Smithers, the global demand for flexible packaging is projected to reach $283 billion in 2022, with an annual growing rate of 4.3% [1]. However, food and cosmetic products wrapped in single servings are an enormous waste of packaging material and thus of resources.

Currently, about 17% of the world’s film production is based on multilayer films [2]. These films are composed of different materials, which can be polymeric (thermoplastics) or non-polymeric (paper or aluminum foils). It is common to produce packaging films consisting of 2 to 17 different layers, each with very specific properties, in order to be able to meet all packaging requirements in total. Since it is currently not possible to effectively recycle such multi-material films, they are usually eliminated from the recycling waste stream and sent to incineration or landfill [3]. In addition, plastic waste ends up in rivers, fields, oceans, and in the end even in the groundwater worldwide. This represents a global environmental problem [4,5]. These plastics practically do not degrade in the environment but accumulate [6]. Over the course of many years, the plastic breaks down into tiny particles called microplastics, which are now found almost everywhere, including our food chain [7]. Microplastics have effects on organisms, humans and ecosystems, the extent of which cannot yet be estimated [6]. The ingestion of microplastics or other hazardous plastic compounds by humans has already been proven. However, once microplastics and additives, etc., are ingested into the human body, their fate and effects are still poorly understood [8]. Microplastics smaller than 20 µm should be able to penetrate organs, and those around 10 µm in size should be able to enter all organs, cross cell membranes, the blood–brain barrier and enter the placenta [9]. There is not enough information available at this time to fully understand the exact effects of microplastics and of other plastic compounds on human health. To overcome this potential hazard to people, living creatures and our environment, it is crucial to have a closer look at the impact of packaging materials and significantly increase recycling and composting rates with less impact on the environs. The use of monomaterial films for packaging, either fossil-based or bio-based, is the most promising way. This may be the key to less plastic pollution in our environment. However, in general, substrate materials alone do not meet the required properties and especially the barrier values to ensure the minimum shelf life of food [10]. 

There are also serious limitations to biodegradable carrier materials such as regenerated cellulose and blends based on polylactic acid and starch, which impede their use in significant amounts. They are often mechanically unstable and do not meet the barrier properties for water vapor or oxygen required for food packaging [11]. However, the use of thin functional coatings can provide a remedy. Good recyclability can be achieved if the coated carrier film does not contain more than 5% foreign material, for example, a coating [12]. In principle, fossil-based, recyclable polyolefin or PET films or bio-based, recyclable films such as PLA or paper and cardboard can be used as substrate materials. If composting is the means of choice, a prerequisite for good compostability of the overall packaging material is that both the substrate material and the functional coating are compostable.

For more than 30 years, the Fraunhofer Institute for Silicate Research ISC in Germany has been developing high-barrier coatings based on inorganic-organic hybrid polymers (ORMOCER^®^). This class of materials combines the properties of organic polymers with those of glassy materials. Such nanocomposites have strong covalent bonds between the inorganic and organic phases, which form a dense network. These materials exhibit excellent barrier properties to gases, water vapor, and flavors and can therefore be used as coatings in food packaging [13]. Nevertheless, the ORMOCER^®^-based coatings are not biodegradable. To enable biodegradable packaging materials as well, the coatings had to be modified for biodegradability while retaining the barrier and other important functional properties. 

The development of biodegradable hybrid polymer layers (bioORMOCER^®^) was achieved through the incorporation and stable linking of chemically modified biopolymers in ORMOCER^®^ matrices [14,15]. The ORMOCER^®^ and the bioORMOCER^®^ coatings exhibit very low barrier values for oxygen and water vapor, they have very good adhesive properties on various substrate materials (different polymers, paper and cardboard) and already show their good properties in very thin layer thicknesses (<1–4 µm). Therefore, packaging solutions with foreign matter contents of less than 5% can be realized and make recycling possible [12]. 

The aim of the recent developments was to further improve the barrier properties of bioORMOCER^®^ coatings. Therefore, hemicellulose was used as the bio-component, as it can be obtained from biological waste streams, for example, from fruit pomace. To achieve covalent linking to the hybrid ORMOCER^®^ matrices, the hemicellulose was chemically modified in a two-step amination reaction. Spectroscopic techniques were used to detect the reaction. The aminated hemicellulose was then used as a new precursor for the syntheses of the functional barrier lacquers. The lacquers obtained were applied to suitable substrate films (e.g., polyolefin films). To achieve the best possible barrier properties also for the new bioORMOCER^®^-based coating material, it was important to realize maximum inorganic and organic crosslinking degrees [13]. The cured coatings were therefore analyzed for their degrees of crosslinking using solid-state NMR spectroscopy. To complete the requirements for packaging applications, the coated film substrates were evaluated with respect to their compostability (according to EN 13432:2000 standard [16]) and barrier properties.

## 2. Materials and Methods 

The tamarind seed gum polysaccharide Glyate^®^ was obtained from DSP Gokyo Food and Chemical Co., Ltd., Osaka, Japan. All reagents were of analytical grade and used without pretreatment or purification. The film substrates were acquired from MI-PLAST Ltd. (PLA/PBAT), Taghleef Industries GmbH (PLA) and state-of-the-art polyolefin film (PO, 200 µm).

### 2.1. Tosylation of the Tamarind Hemicellulose Glyate^®^

The tosylation and amination of the hemicellulose Glyate^®^ were carried out based on the functionalization of cellulose of Schmidt et al. [17]. For dissolution, 10.00 g of Glyate^®^ were taken in a 1000 mL flask. 300 mL of deionized water, 26.65 g (666 mmol) sodium hydroxide and 40 g (666 mmol) urea were added under stirring. The mixture was cooled to −18 °C in a freezer and afterward stirred vigorously at ambient temperature to obtain a transparent Glyate^®^ solution. The solution was cooled to 0 °C and the tosylation was performed by adding 105.85 g (555 mmol) p-toluenesulfonyl chloride (TosCl) and 37.5 mL polyethylene glycol alkyl-(C11–C15) ether (Imbentin). The mixture was vigorously stirred at 0 °C for 24 h and precipitated into 1 L 80% (*v*/*v*) ethanol. The product was separated by filtration, washed three times with 500 mL ethanol, and dried at 50 °C in an oven.

Yield: 20.35 g white powder.

Elemental analysis: C 50.04%, H 5.04%, N 0%, S 11.29%.

FT-IR (KBr, cm^−1^): 1598 (*ν*C=C_aromatic_), 1364 (*ν*_as_SO_2_), 1177 (*ν*_s_SO_2_), 812 (*ν*S–O–C).

### 2.2. Amination of the Tosylated Glyate^®^

For amination, 3.0 g of tosylated Glyate^®^ were added to 32 mL dimethyl sulfoxide (DMSO). The mixture was heated to 100 °C and stirred until dissolution. While stirring, 14.2 g (236 mmol) of ethylendiamine (EDA) were added and the mixture was refluxed for 6 h at 100 °C. After cooling to room temperature, the product was precipitated in 350 mL acetone, washed three times with 100 mL isopropanol and three times with 100 mL acetone. For lacquer synthesis, the moist product was dissolved in deionized water. The remaining solvent was evaporated under reduced pressure. For elemental analysis and NMR, part of the product was dried at 130 °C in an oven for 2 h.

Yield: 28.0 g of a 3.4 wt.-% yellow aqueous solution (pH 9.2).

Elemental analysis: C 47.58%, H 6.87%, N 8.71%, S 4.18%.

FT-IR (KBr, cm^−1^): 1611 (νC=C_aromatic_), 1382 (ν_as_SO_2_), 1175 (ν_s_SO_2_), 809 (νS–O–C).

### 2.3. ORMOCER^®^ and bioORMOCER^®^-Based Lacquers: Synthesis and Coating

ORMOCER^®^ lacquers belong to a material class that has been synthesized by the Fraunhofer Institut für Silicatforschung for several decades now [18,19]. Their synthesis is based on the carefully controlled hydrolysis and condensation reactions of organic element precursors such as organically modified alkoxysilanes. For the ORMOCER^®^ lacquer used in this work, (3-glycidyloxypropyl)trimethoxysilane (GLYEO), tetraethoxysilane (TEOS), aluminum chlorohydrate and acetylacetone stabilized zirconium complexes were employed as educts. bioORMOCER^®^ lacquers were achieved by incorporating EDA-Glyate^®^ into ORMOCER^®^ and adjusting the pH-value to 4.0–4.5 with concentrated HCl. The resulting bioORMOCER^®^ contained 10 wt.-% aminated hemicellulose regarding the solid content of the lacquer. 

The lab-scale coating on films was applied by using a spiral film applicator (Coatmaster 509 MC, ERICHSEN GmbH & Co. KG, Hemer, Germany) with a drawing speed of 12.5 mm/s and a spiral (Model 358, ERICHSEN GmbH & Co. KG, Hemer, Germany) for 20 µm film thickness. Coatings on glass slides were applied by dip-coating with a speed of 1.67 mm/s. Films were then surface activated (Arcotec GmbH, Mönsheim, Germany, coronagenerator, 50% speed, 0.6 kW). The curing of the coatings was accomplished, depending on the used substrates, at 130 °C for 2 h (non-biodegradable substrates) or 80 °C for 2 h (PLA, PLA/PBAT).

On a pilot scale, coatings were applied by MI-PLAST Ltd. using Uteco flexographic off-line printing units (Moda Meccanica srl, Fossò, Italy) with an additional flexographic line Omet type horizontal 400 mm with cooling and drying (UV, IR). The speed was set between 10 and 80 m/min and the coating thickness was approximately 3–4 g/m^2^.

### 2.4. Measurements

#### 2.4.1. FT-IR Spectroscopy

FT-IR transmission spectra were recorded with a Nicolet 6700 infrared spectrometer from Thermo Fisher Scientific, Waltham, Massachusetts, USA, which were carried out using 200 scans and a resolution of 4 cm^−1^ in the range from 4000 to 400 cm^−1^. The samples were measured as KBr pellets (100 g KBr, 10 mg sample). 

#### 2.4.2. Raman Spectroscopy

For the Raman spectra, the RFS 100 spectrometer from Bruker, Billerica, Massachusetts, USA with a power of 500 mW and a measuring range of 4000 to 200 cm^−1^ and a resolution of 4 cm^−1^ was used. The number of scans was between 100 and 500, depending on the sample.

#### 2.4.3. Solid-State-NMR Spectroscopy

The solid-state NMR spectra were recorded at the Julius-Maximilians University of Würzburg and the University of Freiberg. The spectra were recorded on a Avance III HD 400 MHz WB NMR spectrometer from Bruker, Billerica, Massachusetts, USA (^1^H: 400.30 MHz, ^13^C: 100.67 MHz, ^29^Si: 79.52 MHz) using a 4mm triple resonance DVT MAS probe and a 7mm double resonance DVT MAS probe. The chemical shifts are reported relative to tetramethylsilane (TMS) using adamantane for ^13^C and octakis-(trimethylsiloxy)silsesquioxane (Q8M8) for ^29^Si as a secondary standard. ^1^H-^13^C CP experiments were performed with a contact time of 1–2 ms and a 70% ramp. A ZrO_2_ rotor was spun at 5–10 kHz. The recycling delay was set to 3 s. The ^1^H-^29^Si CP measurement was performed with a contact time of 3 ms and an 80% ramp. A ZrO_2_ rotor was spun at 5 kHz. The recycling delay was set to 3 ms. 

#### 2.4.4. Elemental Analysis

The elemental analyses were carried out at the University of Würzburg on an Vario Micro cube from Elementar, Langenselbold, Germany. Calculations of the degree of substitution (DS) are based on the work of Gericke and Heinze [20].

#### 2.4.5. Adhesion Test

The adhesion was determined by means of a modified tape test derived from the cross-cut technique with an adhesive tear-off (DIN EN ISO 2409). For the test, an adhesive tape with a defined bond strength (tesa^®^ Strapping 4287) was stuck onto the coating without bubbles and pressed on. After a period of 1 min, the adhesive tape was peeled off within one second at an angle of about 60° to the direction of pull, and the adhesion was then assessed.

#### 2.4.6. Laser Scanning Microscopy

Laser scanning microscopy (LSM) was performed with a Keyence Deutschland GmbH, Neu-Isenburg, Germany microscope, model VK-X200 series with a violet laser (408 nm).

#### 2.4.7. Hardness and E-Module Measurements

The Martens hardness (HM) and E-Module were determined with the Fischerscope H100 hardness measuring device from Helmut Fischer GmbH, Sindelfingen, Germany. The test force was 10 mN, and the depth resolution was 0.5 nm. A Berkovich diamond was used as the indenter. The measurements were computer-controlled and were evaluated with the aid of H100V-HCU (Helmut Fischer GmbH, Sindelfingen, Germany). The maximum penetration depth was limited by the sample thickness, as it must not exceed 10% of the total layer thickness, and was usually at 0.3 μm. The coatings were measured on glass slides and at least four measurements per sample were recorded. The coating thickness was determined on an alpha-step 200 profilometer from Tencor Instruments. 

#### 2.4.8. Oxygen Transmission Rates Measurement

The oxygen transmission rates (OTR) were measured using the isostatic carrier gas method according to DIN 53380-3 at a relative humidity of 50% and a temperature of 23 °C using the Mocon Ox-Tran 2/21 oxygen permeation measuring device (AMETEK GmbH, Neuwied, Germany). A duplicate determination was carried out for each sample.

#### 2.4.9. Compostability

Compostability (EN 13432:2000) [16]: This European Standard specifies requirements and procedures to determine the compostability and anaerobic treatability of packaging materials by addressing four characteristics: (1) biodegradability; (2) disintegration during biological treatment; (3) effect on the biological treatment process; (4) effect on the quality of the resulting compost. Each specific experimental test is described.

#### 2.4.10. Biodegradability

Biodegradability (ISO 14855-1:2012) [21]: The coated films were measured by metabolic conversion of the plastic material to carbon dioxide to at least 90% in less than six months. The aerobic biodegradability test of materials was carried out with microcrystalline cellulose as a reference material. The composting inoculum was a mature compost derived from an organic fraction of municipal solid waste. The compost was sieved to remove particles over 5 mm in size, and the fine fraction was then used as the inoculum. Control reactors contained only this inoculum without test material. In the composting reactors, 60 g of reference or test material was mixed with 1000 g of inoculum. The reactors were placed in an incubator without light at 58 ± 2 °C and continuously aerated. During biodegradation, microorganisms present in the inoculum converted carbon in the reference or test material into CO_2_. The biodegradation was determined as the percentage of the carbon in the starting reference or test material that was converted into CO_2_. The tests were stopped after 90 days of incubation, when biodegradation reached more than 90%.

#### 2.4.11. Disintegration

Disintegration (ISO 16929:2019) [22]: The film test materials (reduced in size 10 cm × 10 cm) were mixed in a precise concentration with fresh biowaste and introduced in a composting environment after which the biological composting process starts spontaneously. The composting mass was regularly turned over and mixed. The composting process was continued till fully stabilized compost was obtained (after 12 weeks). At the end of the composting process, the compost/test material mixture was sieved over 2 and 10 mm. The mass balance was calculated on the basis of wet weight. The mass of residues above 2 mm has to be less than 10% of the original mass.

#### 2.4.12. Ecotoxicity

Ecotoxicity (OECD 208:2006) [23]: The ecotoxicity assessment was carried out on final composted samples from disintegration tests by comparing the results obtained with the blank compost. The biological test was based on growth assays with two plant species on Control and Test Material compost after disintegration test, mixed to substrate EE0 at 25 and 50% *w*/*w*. Every pot was sown with 100 seeds of Barley or Cress. Three replicates were set up for each series of tests. Pots were incubated in a growth chamber in the following conditions:

- Lighting for 16 h a day at 3000 lx minimum (with light at a wavelength suitable for photosynthesis), at 25 °C temperature and 70% relative humidity;
- Dark phase of 8 h a day, at 20 °C temperature and 80% relative humidity;
- incubation period: 14–21 days from the time when 50% of the control plants have germinated.

At the end of the test, plants were harvested and the following were quantified:

- The number of plants per pot (germination);
- The fresh (wet) weight of biomass per pot;
- The dry weight of biomass per pot (after a drying period of 1 day at 60 °C).

The germination rate and plant biomass of the sample compost of both plant species must be greater than 90% of that of the corresponding blank compost.

#### 2.4.13. Overall Migration

Overall migration as defined by EU Regulation 10/2011: The migration tests were carried out to determine the total amount of non-volatile substances that might migrate into foodstuffs. Simulants were used in accordance with Directives 85/572/EEC, 82/711/EEC, 93/8/EEC and 97/48/EC. The methods are described in European standard series EN 1186 (draft methods at the time of testing) [24]. Test specimens of 1 dm^2^ were immersed in the simulant (100 mL) for a set time at a chosen temperature. At the end of the test period, each specimen was removed from the simulant. The simulants were then evaporated to dryness, and the mass of the non-volatile residue was determined gravimetrically. The list of test conditions and methods is provided in Table 1.

#### 2.4.14. Antimicrobial Properties

Antimicrobial properties: (ISO 22196:2011) [25]: Standardized test organisms were inoculated onto the surface of test material for a period of 24 h: tests were performed on two bacterial species, *Escherichia coli* and *Staphylococcus aureus*, corresponding to 5 × 10^5^ cells/mL. The test was performed on 3 replicates from the treated material and 6 specimens of the untreated material as control samples. In addition, 3 untreated test specimens were used to measure viable cells immediately after inoculation and 3 specimens were used to measure viable cells after incubation for 24 h. After incubation, microbial concentrations were quantified. The relative reduction of microorganisms compared with the initial concentrations and the control surface was calculated. Surviving microorganisms were counted to evaluate the antimicrobial activity of the test material. For the calculation of antibacterial activity, data were expressed as Colony Forming Units (CFU) per cm^2^ (CFU/cm^2^). The antibacterial activity was calculated using the following equation:

R = (U_t_ − U_0_) − (A_t_ − U_0_) = U_t_ − A_t_ (1)

where:

R is the antibacterial activity;

U_0_ is the average of the common logarithm (base 10 logarithm) of the number of viable bacteria, in cells/cm^2^, recovered from the untreated test specimens immediately after inoculation;

U_t_ is the average of the common logarithm of the number of viable bacteria, in cells/cm^2^, recovered from the untreated test specimens after 24 h;

A_t_ is the average of the common logarithm of the number of viable bacteria, in cells/cm^2^, recovered from the treated test specimens after 24 h.

## 3. Results and Discussion

### 3.1. Functionalization of Glyate^®^

#### 3.1.1. Structure and Properties of Glyate^®^

**Figure 1:**
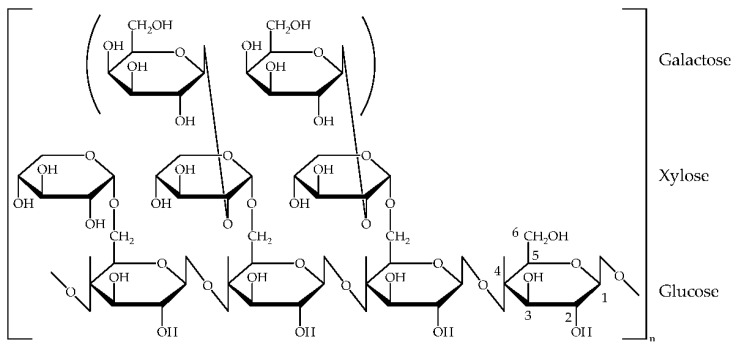
Structure of tamarind seed gum Glyate^®^. The ratio of galactose, xylose and glucose is 1.41:3.18:4.00 [29].

An important step in the realization of a bioORMOCER^®^-based coating material is the adaptation of the biodegradable component to the ORMOCER^®^ basecoat. The bio-component has to be soluble and must possess functional groups capable of forming a covalent bonding to the base lacquer. These requirements may be met by introducing amino groups that promote solubility in aqueous systems and can react with the epoxy moieties of the ORMOCER^®^. Thus, the first aim is the introduction of a good leaving group for a subsequent nucleophilic S_N_ reaction. Tosylation followed by amination is a well-known method in literature to synthesize a water-soluble cellulose. Often, tosylation is carried out in *N*,*N*-Dimethylacetamide (DMA)/Lithium chloride [26,27]. However, the tosylation step is also possible in an environmentally friendly aqueous medium [28]. Based on the tosylation of cellulose, the tamarind seed gum hemicellulose Glyate^®^ could also be functionalized. The chemical structure of tamarind seed gum consists of oligosaccharide unit<s as shown in Figure 1. The hemicellulose possesses a 1,4-β-glucan main chain and three xylose residues branched by α-1,6 linkage for every four glucose residues. Additionally, zero or two galactoses bind to some of the branched xylose residues in each heptasaccharide unit by β-1,2 linkage. The ratio of galactose, xylose, and glucose of the whole tamarind gum polysaccharide is 1.41:3.18:4.00 and its molecular weight is estimated to be about 4,700,004 [29].

#### 3.1.2. Tosylation of Glyate^®^

Taking into account the ratio of saccharides, the average oligosaccharide has a molecular weight of 1298 g/mol and possesses 22.6 free hydroxyl groups. In order to achieve a high degree of substitution, tosylation was performed with an excess amount of *p*-toluenesulfonyl chloride (TosCl). The molar ratio of unbound hydroxyl groups to TosCl was 1:3.19. It has been found by Schmidt et al. that nonionic surfactants like Imbentin are beneficial to the efficiency of cellulose tosylation reaction and also prevent undesired S_N_ substitutions of the tosyl moieties by chloride ions [17]. They also found that a low reaction temperature of 0 °C leads to a higher degree of substitution compared to reaction at room temperature or above. By transferring those results onto the hemicellulose Glyate^®^, a product with a high degree of tosylation was achieved. By elemental analysis it is possible to estimate the degree of substitution DS. Based on the sulfur content of 11.29%, 10.0 tosylate groups are introduced to an oligosaccharide unit with DS calculations according to Gericke and Heinze [20]. Due to sterical reasons, the most reactive hydroxyl group of Glyate^®^ is at position six of glucose and galactose [17,30,31]. Xylose does not possess a primary hydroxyl group. Due to the α-1,6 linkage to xylose, most primary hydroxyl groups in the glucose units are also unavailable for functionalization. Despite this comparatively low number of easily accessible hydroxyl groups, the tosylated Glyate^®^ exhibits a high degree of substitution. This leads to the assumption that less reactive positions like two and three and, in the case of galactose, position four has also been tosylated. The tosylated product is soluble in DMSO and dimethylacetamide, which is an important prerequisite for subsequent functionalization.

**Figure 2:**
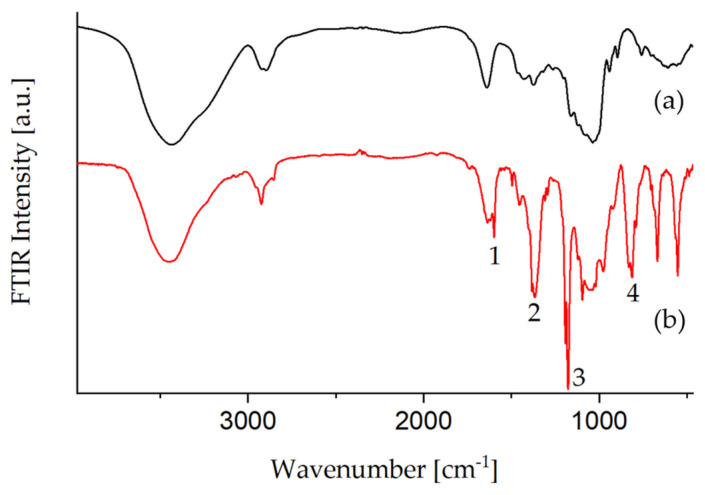
FTIR spectra of (**a**) Glyate^®^ and (**b**) tosylated Glyate^®^.

The FTIR spectra of Glyate^®^ and tosylated Glyate^®^ shown in Figure 2 confirm the successful introduction of tosylate groups. According to Table 2 various bands can be assigned to the sulfonic acid ester group (1364, 1177 and 812 cm^−1^) and the aromatic ring (1598 cm^−1^) [17,28].

#### 3.1.3. Amination of Glyate^®^

Amination was carried out with excess EDA. The product was always kept moist during reprocessing and stored as an aqueous solution to avoid hornification upon drying [32]. Elemental analysis yielded a sulfur content of 4.18% and a nitrogen content of 8.71%. Calculations according to Gericke and Heinze based on the nitrogen and sulfur content yield a DS_EDA_ = 6.0 and DS_Tos_ = 2.5 per polysaccharide unit [20]. The sulfur content indicates remaining tosylate groups. This is not surprising, because it has been found for cellulose that the S_N_ amination reaction is even more regioselective than the tosylation and almost exclusively occurs at the C6 atom [33]. Position 2 and 3 are sterically less accessible and not sufficiently reactive for the nucleophilic S_N_ reaction. Therefore, secondary tosylates remain on the newly formed amino derivatives. Similarly, tosylates in less reactive positions remain on the polysaccharide for the hemicellulose Glyate^®^. However, the reactivity of Glyate^®^ differs from that of cellulose. On average, glucose and galactose provide only max. 2.23 primary available C6 positions in the polysaccharide. Nevertheless, DS_EDA_ = 6.0 was achieved. This high DS strongly indicates a deviant reactivity of Glyate^®^ from cellulose, as some C2, C3 or C4 positions must have been aminated. A possible explanation is that the hydrogen bonds between the different saccharides may be weaker than in cellulose due to steric influences of the polysaccharide leading to higher reactivity of some secondary hydroxyl groups. Another reason for the high DS_EDA_ may be the alkaline conditions during tosylation. In contrast to cellulose, which is generally stable against alkaline hydrolysis, hemicelluloses are easier to hydrolyze. The ether bonds present in Glyate^®^ are more easily hydrolyzed under acidic conditions, but it is possible that the alkaline pretreatment led to additional reactive hydroxyl groups [34,35]. The high degree of amination makes a covalent bonding to ORMOCER^®^-matrix possible and improves the water solubility. On the other hand, remaining tosylate moieties are hydrophobic. Thus, it is desirable to minimize the remaining tosylate moieties. However, starting the amination with a Glyate^®^ containing fewer tosylate groups yielded a product with a lower DS_EDA_, which was not water-soluble. Therefore, an appropriate balance of both functional groups must be adjusted.

**Figure 3:**
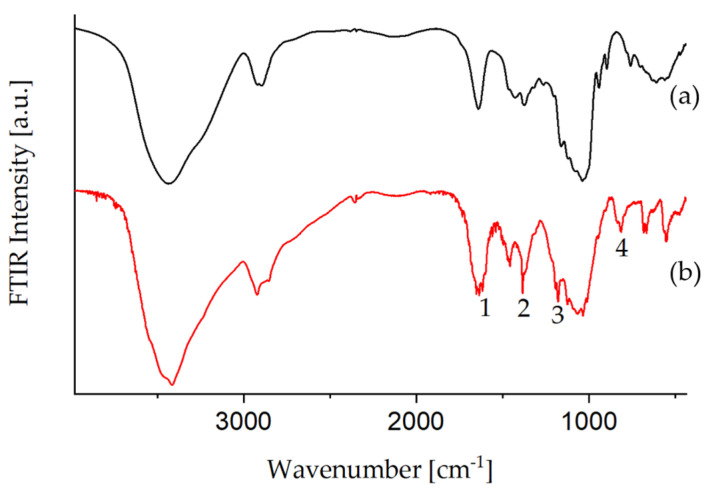
FTIR spectra of (**a**) Glyate^®^ and (**b**) EDA-Glyate^®^.

Figure 3 shows the FTIR spectrum of EDA-Glyate^®^. As expected, bands originating from remaining tosylate groups are observed (bands 1–4). However, the intensity of those signals is strongly diminished after amination. This indicates tosylates leaving the polysaccharide in an S_N_ reaction, which are replaced by either EDA or hydroxyl moieties.

**Figure 4:**
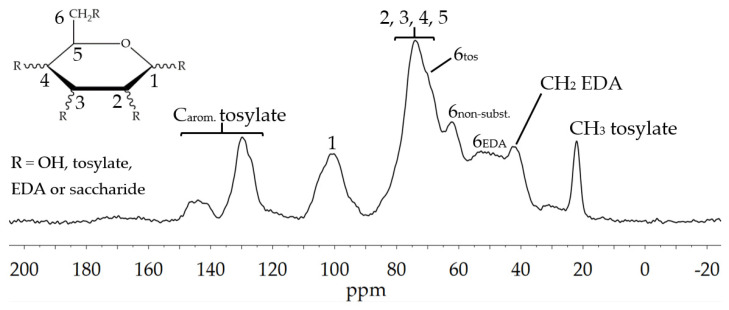
^13^C solid-state NMR spectrum of EDA-Glyate^®^.

The solid state NMR in Figure 4 also affirms remaining tosylate groups with aromatic signals in the region 120–150 ppm and primary CH_3_-groups at 21 ppm. Furthermore, the different substitution states at the C6 position can be observed. A shoulder at 69 ppm indicates C6_tosylated_, the signal at 61 ppm C6_non-substituted_ and the peaks in the region around 50 ppm represent aminated carbons. Additionally, the CH_2_-signal of EDA can be found at 42 ppm, proving the successful amination [17].

### 3.2. Development of Barrier Coatings with EDA-Glyate^®^

The synthesized EDA-Glyate^®^ can impart new properties to existing ORMOCER^®^ formulations, especially with regard to biodegradability. The idea is that the introduced biocomponents in the ORMOCER^®^-matrix act as predetermined breaking points during biodegradation. The addition of nitrogen or oxygen favors the biodegradability of the ORMOCER^®^ matrix, since heteroatom-containing polymers are often more easily degraded, for example, by hydrolyzing enzymes [36].

For the successful integration of the aminated polysaccharide into the ORMOCER^®^ matrix, solubility is a crucial factor. ORMOCER^®^ is a sol-gel based material class. Thus, alcohol is formed during synthesis. Therefore, the functionalized polysaccharide has to be soluble in an aqueous-alcoholic medium. Solubility in aqueous or alcoholic systems is often an issue with cellulose and related polysaccharides. In the case of functionalized Glyate^®^, the water solubility is improved by the high degree of amination. Furthermore, the EDA-Glyate^®^ does not precipitate when ethanol is formed due to hydrolysis of silanes. The ORMOCER^®^ formulation used in this publication was chosen as the base system because of its high water content compared to other compositions. The solvent system in the finished coating contains water and ethanol in a weight ratio of about 1:1.4.

#### 3.2.1. bioORMOCER^®^ Synthesis 

**Figure 5:**
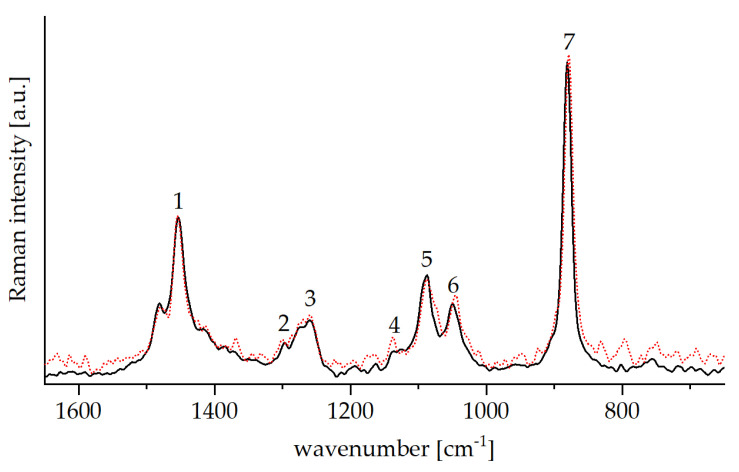
Raman spectra of liquid ORMOCER^®^ base lacquer (black) and EDA-Glyate^®^ containing bioORMOCER^®^ lacquer (red, dotted). Peak assignment is shown in Table 3. The band at 1259 cm^−1^ denotes the closed epoxide ring.

ORMOCER^®^ and bioORMOCER^®^ are hybrid materials possessing an inorganic and an organic network. Both networks combine to make the hybrid material an excellent barrier coating for packaging. While the organic network provides flexibility, the inorganic network lends durability and plays a major role in the barrier performance. Raman spectroscopy is a convenient method for lacquer characterization and quality assurance, as it allows to check if the requirements for efficient network formation are met. The Raman spectra of the liquid ORMOCER^®^ and EDA-Glyate^®^ containing bioORMOCER^®^ are shown in Figure 5 and the bands are assigned in Table 3. 

The inorganic network of the hybrid material is formed via the sol-gel process. For the condensation of the silanes TEOS and GLYEO a prior hydrolysis of the trialkoxy moieties is essential. Those trialkoxy moieties show a distinct Si-O symmetric breathing peak at about 650 cm^−1^ in the Raman spectrum, which vanishes during hydrolysis [37]. Another indication of the hydrolysis is the formation of ethanol. The reaction can be followed by the increase of the free ethanol band at 880 cm^−1^, which reaches a maximum when the hydrolysis is complete.

**Figure 6:**
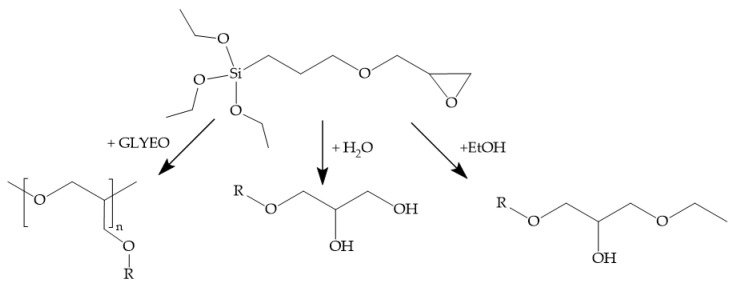
Scheme of possible reactions of 3-glycidyloxypropyl)trimethoxysilane (GLYEO) [38].

The organic network originates from the polymerization of the epoxy groups of GLYEO. The organic network is formed during the thermal curing process, and premature ring opening of the epoxy groups leads to undesired byproducts like diols and methylether, as shown in Figure 6 [38]. Those byproducts do not partake in the organic network formation and are detrimental to the barrier performance.

The band No. 3 of the closed epoxy ring breathing shows at 1259 cm^−1^ in the Raman spectrum.

Both ORMOCER^®^ and bioORMOCER^®^ spectra display the signs for a successful hydrolysis as well as intact epoxy rings. It should also be noted that no clear signals from EDA-Glyate are observed. This is due to the relatively low concentration and the overlapping bands of the Raman–intensive ORMOCER^®^ component signals.

#### 3.2.2. Physical-Chemical Properties of the Cured bioORMOCER^®^ Coatings

The ORMOCER^®^ and bioORMOCER^®^ lacquers are coated on different substrates (PLA, PLA/PBAT, polyolefine, glass) and thermally cured. The inorganic network is finalized by the curing process and the organic network forms.

**Figure 7:**
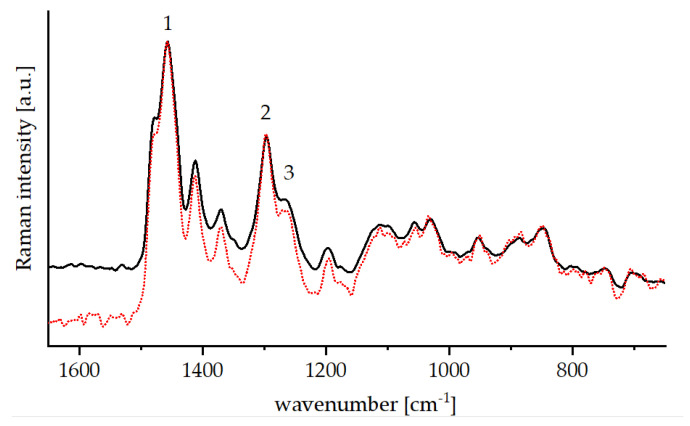
Raman spectra of cured ORMOCER^®^ coating (black) and EDA-Glyate^®^ containing bioORMOCER^®^ coating (red, dotted).

The Raman spectra of the cured lacquers in Figure 7 show no ethanol bands as the alcohol is evaporated during the thermal curing. The spectra display prominent bands of CH_2_- and CH_3_-groups in the cured lacquers as well as a significant reduction of signal 3, which indicates a closed epoxide ring. This means most epoxide groups in both the ORMOCER^®^ and bioORMOCER^®^ opened during the thermal treatment and were able to participate in forming the organic network by polymerization. Regarding the inorganic network formation, many overlapping signals adhering to partially hydrolyzed and condensed silanes can be observed in the lower wavenumber region of the Raman spectrum. Partially hydrolyzed species can typically be found in the range of 645–720 cm^−1^, while Si–O–Si vibrations belonging to silica dimers or networks show at 795–830 cm^−1^ [41]. Further Si–O–Si stretching bands can be found at about 845 cm^−1^, 1050 cm^−1^ and generally in the spectral range between 1000 and 1300 cm^−1^ [42,43]. These indicate that the thermal curing was effective and that a silane network, or at least siloxane structures, were formed.

**Figure 8:**
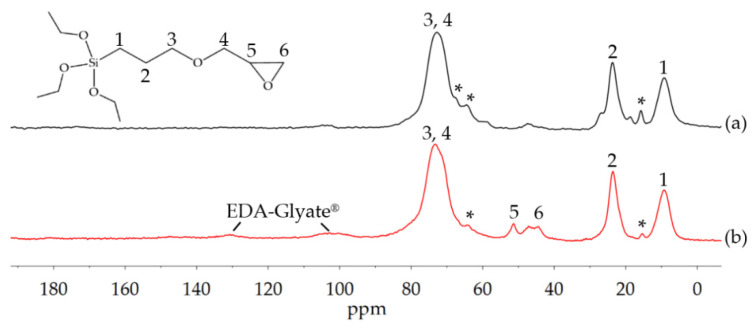
^13^C solid-state NMR spectra of (**a**) cured ORMOCER^®^ coating and (**b**) EDA-Glyate^®^ containing bioORMOCER^®^ coating. Asterisks denote epoxy ring opening side products.

Solid-state-NMR is another useful method for determining the network formation. The ^13^C-NMR spectra of the cured ORMOCER^®^ and bioORMOCER^®^ are shown in Figure 8. The organic network is formed by the polymerization of GLYEO. Therefore, there should be little to no signal of closed epoxy rings in the spectra of the cured lacquers. The intact epoxy ring leads to signals at 44 and 51 ppm, which disappear during ring opening. The ^13^C-NMR-spectrum of the ORMOCER^®^ shows no epoxy ring peaks. In the spectrum of the bioORMOCER^®^ however, epoxy ring signals can be observed. Interestingly, there are also more pronounced signals of side products of the ring opening in the ORMOCER^®^ spectrum. Ether and diol side products, as shown in Figure 6, can be found at 16, 64 and 66 ppm. While the epoxy ring opening of the ORMOCER^®^ is more complete, the side product formation is also higher than in the bioORMOCER^®^.

**Figure 9:**
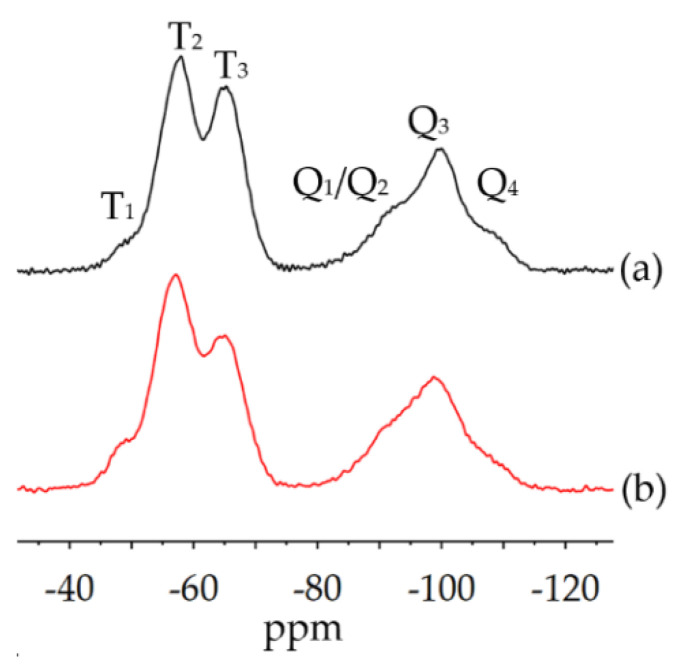
^29^Si solid-state NMR spectra of (**a**) cured ORMOCER^®^ and (**b**) cured bioORMOCER^®^.

The inorganic network formation of ORMOCER^®^ and bioORMOCER^®^ can be analyzed by the ^29^Si-spectra in Figure 9. T_x_-groups indicate the state of GLYEO and Q_x_-groups are derived from TEOS. A higher subscript means a higher degree of condensation. The network formation in both coatings is generally comparable, with the bioORMOCER^®^ displaying a slightly lower network density. The bioORMOCER^®^ shows a lower T_3_/T_2_-ratio indicating less condensed GLYEO. Furthermore, a shoulder at 90–95 ppm indicates less connected Q-groups.

**Figure 10:**
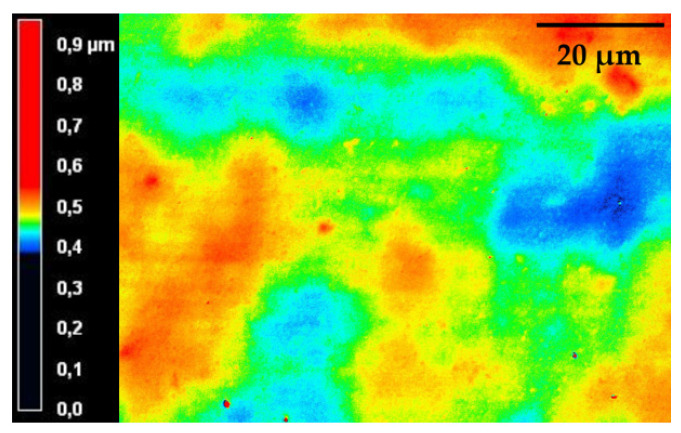
Laser scanning microscopy (LSM) picture of a cured bioORMOCER^®^ coating. The different colors represent the height profile of the surface.

For application as packaging material, good adhesion on the substrate is crucial. The adhesion of the coatings on the different substrates was examined via a modified tape-test without cross-hatching. Both ORMOCER^®^ and bioORMOCER^®^ showed very good adhesion on all tested substrates. The applied coatings are generally very thin with a coating weight of 2–4 g/m^2^. This ensures flexibility, keeps the costs low and enables monomaterial packaging, as a recyclable monomaterial may only include up to 5% foreign compounds [12]. In addition, ORMOCER^®^ coatings typically possess very smooth surfaces and can also act as a planarization layer [15]. bioORMOCER^®^ coatings also possess smooth surfaces, as shown in the LSM picture in Figure 10. Ra measurements on four different samples gave Ra values of 15.7–17.0 nm. Ra is the arithmetic average of the absolute values of the profile height deviations from the mean and is a standard parameter for describing the surface roughness. The measured values prove the low surface roughness of the bioORMOCER^®^.

An additional interesting property of the lacquers is the universal hardness or Martens hardness (HM). The hardness is affected by the network density. A denser network usually leads to harder materials. The HM as well as the E-Module of the cured lacquers on glass slides are shown in Table 4. The ORMOCER^®^ is significantly harder and possesses a higher E-Module than the corresponding EDA-Glyate^®^ containing bioORMOCER^®^. This difference is not surprising because, as already shown, the incorporation of the relatively large hemicellulose reduces the inorganic and organic network density. However, the hardness and E-Module are still higher than many commonly used barrier coatings, for example, based on EVOH [44].

**Figure 11:**
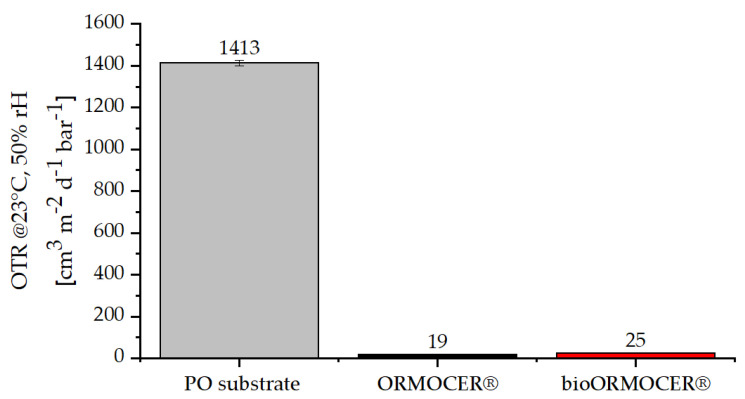
Oxygen transmission rates (OTR) of PO substrate and PO coated with ORMOCER^®^ and bioORMOCER^®^.

The incorporation of EDA-Glyate^®^ into ORMOCER^®^ leads to a functional barrier lacquer despite decreased network density. This is clearly shown in the oxygen transmission rates (OTR) displayed in Figure 11. For application as packaging, low OTR values are essential to protect sensitive goods from oxidation and thus keep foods fresh. The uncoated PO substrate has an OTR of 1413 cm^3^ m^−2^ d^−1^ bar^−1^. This value can be massively reduced to 19 cm^3^ m^−2^ d^−1^ bar^−1^ by applying a thin layer of about 3 µm of ORMOCER^®^. With an OTR of 25 cm^3^ m^−2^ d^−1^ bar^−1^, the bioORMOCER^®^ coating provides an only slightly worse value, still low enough for numerous packaging applications [9].

### 3.3. Evaluation of the Compostability, Antimicrobial Properties and Migration of Cured bioORMOCER^®^ Coatings

The compostability, migration and antimicrobial properties of bioORMOCER^®^ were tested on biodegradable substrates. To evaluate the compostability, the bioORMOCER^®^ coating was tested for biodegradation, disintegration and ecotoxictity. As larger than lab quantities were needed, the bioORMOCER^®^ lacquer was applied using a roll-to-roll system. The samples tested were PLA + bioORMOCER^®^ with an average overall thickness of 45 µm and PLA/PBAT + bioORMOCER^®^ with a thickness of 75 µm, while the coating thickness was 2–4 µm.

#### 3.3.1. Biodegradability

**Figure 12:**
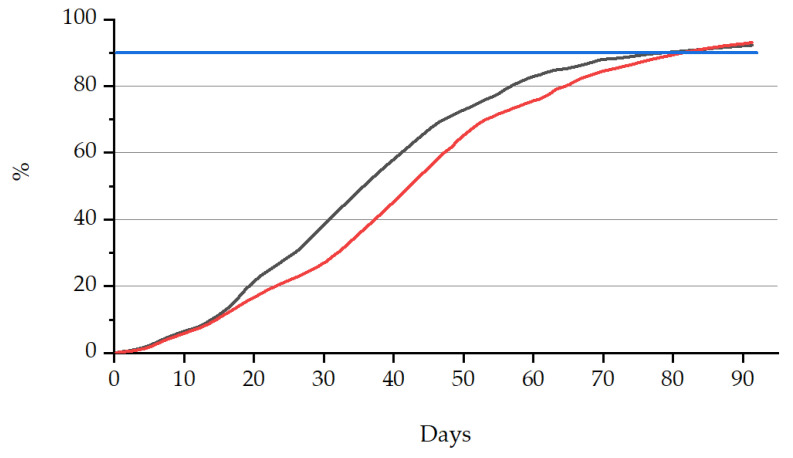
Biodegradability graphs for tested samples with PLA + bioORMOCER^®^ (black), PLA/PBAT + bioORMOCER^®^ (red) and the blue line representing the target value.

According to European Standard EN 13,432, a material can be called biodegradable when the biodegradation is at least 90% in total after a plateau has been reached. The standard prescribes a test duration maximum of 6 months. The average biodegradation percentages and the time lapse of biodegradability percentages are provided in Table 5 and Figure 12. Both samples are more than 90% biodegradable within 6 months, meaning that more than 90% of the organic carbon of the test material is converted into carbon dioxide by microorganisms under industrial composting conditions.

The quality of the inoculum used for the test is confirmed by the background average activity, which is 78 mg CO_2_/g volatile solids (VS) after 10 days of incubation (production of 50–150 mg CO_2_/g vs. should be measured as background during the first 10 days).

The results demonstrated that both bioORMOCER^®^ films fulfilled the requirements for biodegradation and can be described as biodegradable under controlled composting conditions. Significantly, comparing the biodegradation percentage of linear PLA and the experimental bioORMOCER^®^ films, percentages are similar, meaning that no significant effect is detectable for the presence of the coating [45].

#### 3.3.2. Disintegration

According to European Standard EN 13432, a material can be called disintegrable when the disintegrated material below 2 mm sieve is at least 90% in total after 12 weeks. For disintegration testing, the residual material is assembled on a 2 mm sieve, separated and weighed. The disintegration degree is quantified. The obtained results are shown in Table 6 (final values). Both samples contain no pieces larger than 2 mm and are therefore 100% disintegrable within 12 weeks. According to the obtained percentages, the analyzed samples comply with the requirements of EN 13,432 for the disintegration test and do not produce any visual contamination at the end of the disintegration test.

#### 3.3.3. Ecotoxicity

Biodegradable packaging materials should not produce negative ecotoxic effects. This is verified by a plant growth test with Barley and Cress on the final compost. There must be no difference between the test compost and the control compost in terms of germination and biomass growth. According to point A4 of Annex A of EN 13,432 standard, the germination rate and the plant biomass of both plant species, at each dilution tested, must be higher than 90% or equal to that of the control test. The ecotoxicity test is applied on the mixture of the final composted residues from the coated film samples, called “MIX+ bioORMOCER^®^” to evaluate a possible ecotoxic effect of the bioORMOCER^®^ coating. The experimental data of the toxicity tests performed on barley are given in Table 7 and on cress are reported in Table 8. The percentages (at two different levels, 25% and 50%) and the consistent weighted grams of added final composted residues, the amount of used substrate (standard soil), the number of germinated seeds (over a total of 100 used seeds) and the final fresh weight of biomass are reported in both tables.

Average values of germinated plants, fresh biomass of the plants and relative standard deviation calculated for barley and cress are reported in Table 9.

Percent emergence as compared to controls (germination rate as ratio between sample and control values of germination) calculated for barley and cress are reported in Table 10. The results show that the germination rate and fresh weight of the sample compost for both plant species are greater than 90% of the corresponding blank compost (control).

No significant differences are observed with regard to the germination between the control compost and the test compost at both concentration levels. The samples coated with bioORMOCER^®^ showed no phytotoxic effect with all values greater than 90% compared with the blank compost.

#### 3.3.4. Antimicrobial Properties

The following tables show the results obtained by the antimicrobial activity test employing bacterial concentration according to the ISO 22196:2011 method. The data are the average values of three replicate tests executed for every specimen, each one performed in duplicate for every bacterial strain. In Table 11, the results obtained on uncoated and coated PO are presented. Table 12 reports antibacterial activity (R) calculated for the tested samples.

After the evaluation of the antimicrobial properties of antibacterial activity of the bioORMOCER^®^ coating, both samples showed an effective activity on *Escherichia coli* and *Staphylococcus aureus* with comparable results.

#### 3.3.5. Overall Migration

When the packaging comes into contact with food, it is essential to know if any substances migrate out of the material. The food contact is simulated with different model substances. The total migration tests of the packaging are allowed to proceed for 10 days at 60 °C. This serves as “worst condition” and simulates a long-lasting contact of food at room temperature.

Hence, packaging samples with negative results at the previous conditions are additionally tested in less rigorous conditions (10 days at 40 °C). Coated and uncoated PO and PLA films are analyzed for migration at 60 °C after 10 days of contact: the results for the total migration of specimens are presented in Table 13. Overall migration exceeding 10 mg/dm^2^ is not in compliance with the EU Regulation 10/2011 for food contact application.

The application of bioORMOCER^®^ coating provides very good results as they comply with the official limit for almost all tested samples with all the simulants. In particular, the migration results with water and ethanol simulants are improved, especially for the PO substrate treated with the coating. 

If a bioORMOCER^®^ coating is applied on a biodegradable film, only one test (PLA + bioORMOCER^®^) overcomes the limit with acetic acid used as a simulant. Nevertheless, the result is comparable with the limit, which means that only small improvements in terms of the efficacy of the application will be needed to reach the overall compliance. All other tested conditions provide results in compliance with the limit.

The excellent values of the coated PO samples against water and ethanol in comparison to the coated biodegradable films may be explained by the curing temperatures. Coatings on PO can be cured at 130 °C, whereas on the PLA quality used, curing is possible only at 80 °C. While both curing conditions are sufficient for effective network formation, coatings cured at higher temperatures are generally somewhat more stable.

The biodegradable film that provided a negative result at 60 °C was tested again at 40 °C to assess if lower temperatures could decrease the experimental values for migration: the results shown in Table 14 are in compliance with the limit at the lower temperature.

## 4. Conclusions

In order to synthesize new biodegradable, functional coatings, the tamarind hemicellulose Glyate^®^ was successfully functionalized with EDA. A sufficiently high DS was achieved to ensure compatibility with the ORMOCER^®^ base system. The functionalization and integration of Glyate^®^ is especially interesting as hemicelluloses are a renewable material source and are often agricultural residues, which do therefore not compete with food crops [46]. A transfer of the functionalization with EDA onto other hemicelluloses or biocomponents may be feasible and offer a new range of educts for the development of biodegradable coatings.

The newly synthesized bioORMOCER^®^ coating showed promising properties with compostability and good barrier properties against oxygen. Furthermore, the coating is applied as a thin layer, which enables easily recyclable monomaterial packaging. Modern packagings made of plastic or paper are usually complexly composed of many different materials. Each material in the composite performs a specific task. The different layers cannot be separated easily [47]. This impedes recycling and contributes to littering our environment. Highly functionalized bioORMOCER^®^ coatings will render many such complex packagings obsolete. These new materials can play a major role in the future design and development of new innovative and more environmentally friendly packaging materials.

## Figures and Tables

**Table 1:** List of test conditions and methods for overall migration test.

Simulant	Test Conditions	Overall Migration Test
Distilled water	10 days, 60 °C and 40 °C, total immersion	EN 1186-3
Ethanol 10% *v*/*v*	10 days, 60 °C and 40 °C, total immersion	EN 1186-3
Acetic acid 3% *w*/*v*	10 days, 60 °C and 40 °C, total immersion	EN 1186-3

**Table 2:** FTIR bands of Glyate^®^ and functionalized Glyate^®^, only the most relevant bands are listed. Data from [28].

Nr.	Wavenumber (cm^−1^)	Assignment
1	1598	*ν* (C=C_aromatic_)
2	1364	*ν*_as_(SO_2_)
3	1177	*ν*s (SO_2_)
4	812	*ν* ( S–O–C)

**Table 3:** Raman bands of ORMOCER^®^ and bioORMOCER^®^ (only the most relevant bands are listed). Data from [37,39,40].

No.	Wavenumber (cm^−1^)	Assignment
1	1453	ν_asym_ (CH_3_),ν_asym_ (CH_2_), δ(CH_2_) in ethanol, TEOS, GLYEO
2	1298	τ (CH_2_) in TEOS, GLYEO
3	1259	ν_ip_(Epoxy ring) in GLYEO
4	1136	ν_asym_ (COC) acetylacetone
5	1087	ν_asym_ (CCO) in ethanol
6	1049	ν (CO) in ethanol
7	880	ν_sym_ (CCO) in ethanol

**Table 4:** Martens hardness and E-Module of ORMOCER^®^ and bioORMOCER^®^ measured on glass slides.

Coating	Martens Hardness (HM) (N/mm^2^)	E-Module (GPa)
ORMOCER^®^	406.08 ± 6.16	6.95 ± 0.09
bioORMOCER^®^	287.23 ± 7.51	5.30 ± 0.21

**Table 5:** Final biodegradability percentages for tested samples.

Sample Name	Biodegradability (%)	Time (Elapsed Days)
PLA + bioORMOCER^®^	92.9	91
PLA/PBAT + bioORMOCER^®^	93.0	91
Value limit (EN 13432)	>90	<180

**Table 6:** Disintegration percentages for the tested final samples.

Sample Name	Disintegration (%)
PLA + bioORMOCER^®^	100
PLA/PBAT + bioORMOCER^®^	100
Value limit (EN 13432)	>90 after 12 weeks

**Table 7:** Experimental toxicity data—Barley.

Sample	Sample (%)	Sample (g)	Substrate (g)	Germination (Number)	Fresh Weight (g)
Control compost	25	112.5	337.5	95	40.86
	25	112.5	337.5	97	41.33
	25	112.5	337.5	92	41.00
	50	225	225	92	39.95
	50	225	225	91	38.69
	50	225	225	94	40.43
Test compost MIX+ bioORMOCER^®^	25	112.5	337.5	88	38.32
	25	112.5	337.5	94	37.80
	25	112.5	337.5	90	37.42
	50	225	225	91	36.79
	50	225	225	93	37.14
	50	225	225	96	37.62

**Table 8:** Experimental toxicity data—Cress.

Sample	Sample (%)	Sample (g)	Substrate (g)	Germination (Number)	Fresh Weight (g)
Control compost	25	60	180	94	8.25
	25	60	180	90	7.32
	25	60	180	92	7.90
	50	120	120	89	7.10
	50	120	120	91	7.41
	50	120	120	95	8.09
Test compost MIX+ bioORMOCER^®^	25	60	180	90	7.70
	25	60	180	88	7.80
	25	60	180	86	8.06
	50	120	120	88	7.51
	50	120	120	91	7.64
	50	120	120	91	7.26

**Table 9:** Germination and fresh weight average values for Barley and Cress.

Barley	Concentration (%)	Germination (Number)		Fresh Weight (g)	
		AVG	STD	AVG	STD
Test compost dilution (MIX+ bioORMOCER^®^)	25	90.67	3.0	37.84	0.45
	50	93.33	2.51	37.18	0.41
**Cress**					
Test compost dilution (MIX+ bioORMOCER^®^)	25	88.0	2.0	7.85	0.19
	50	90.0	1.73	7.47	0.19

**Table 10:** Final results on germination rate and fresh weight.

	Concentration (%)	Germination Rate (%)	Fresh Weight (%)
**Barley**			
Test compost dilution (MIX+ bioORMOCER^®^)	25	96.1	92.2
	50	102	93.7
**Cress**			
Test compost dilution (MIX+ bioORMOCER^®^)	25	96.1	100
	50	97.9	99.2

**Table 11:** Bacteria recovery from uncoated and coated films.

	Recovery after Inoculation (cells/cm^2^)		Recovery after 24 h Incubation (cells/cm^2^)	
	*S. aureus*	*E. coli*	*S. aureus*	*E. coli*
Uncoated PO	21,875	21,250	20,625	20,000
PO + bioORMOCER^®^ (Run 1)	-	-	213	1750
PO + bioORMOCER^®^ (Run 2)	-	-	300	2938

**Table 12:** Antibacterial activity of bioORMOCER^®^.

	Antibacterial Activity (R)	
	*S. aureus*	*E. coli*
PO + bioORMOCER^®^ (Run 1)	1.98	1.06
PO + bioORMOCER^®^ (Run 2)	1.83	0.84

**Table 13:** Overall migration results for coated and uncoated samples at 60 °C for 10 days (threshold limit: 10 mg/dm^2^).

	Water (mg/dm^2^)	Ethanol 10% (mg/dm^2^)	Acetic Acid 3% (mg/dm^2^)
PO film	12	15	4.2
PO film + bioORMOCER^®^	<0.1	<0.1	7.7
PLA film	1.7	3.1	3.2
PLA + bioORMOCER^®^	9.5	5.9	11.8
PLA/PBAT + bioORMOCER^®^	5.7	3.1	6.4

**Table 14:** Overall migration results for coated and uncoated samples at 40 °C for 10 days (10 mg/dm^2^, as threshold limit).

	Water (mg/dm^2^)	Ethanol 10% (mg/dm^2^)	Acetic Acid 3% (mg/dm^2^)
PLA + bioORMOCER^®^	6.4	8.8	7.6

## Data Availability

The data presented in this study are available on request from the corresponding author.
